# Monoamine transporters: insights from molecular dynamics simulations

**DOI:** 10.3389/fphar.2015.00235

**Published:** 2015-10-16

**Authors:** Julie Grouleff, Lucy Kate Ladefoged, Heidi Koldsø, Birgit Schiøtt

**Affiliations:** ^1^Center for Insoluble Protein Structures and Interdisciplinary Nanoscience Center, Department of Chemistry, Aarhus UniversityAarhus, Denmark; ^2^Department of Biochemistry, University of OxfordOxford, UK

**Keywords:** molecular dynamics, monoamine transporters, serotonin transporter, dopamine transporter, norepinephrine transporter

## Abstract

The human monoamine transporters (MATs) facilitate the reuptake of the neurotransmitters serotonin, dopamine, and norepinephrine from the synaptic cleft. Imbalance in monoaminergic neurotransmission is linked to various diseases including major depression, attention deficit hyperactivity disorder, schizophrenia, and Parkinson’s disease. Inhibition of the MATs is thus an important strategy for treatment of such diseases. The MATs are sodium-coupled transport proteins belonging to the neurotransmitter/Na^+^ symporter (NSS) family, and the publication of the first high-resolution structure of a NSS family member, the bacterial leucine transporter LeuT, in 2005, proved to be a major stepping stone for understanding this family of transporters. Structural data allows for the use of computational methods to study the MATs, which in turn has led to a number of important discoveries. The process of substrate translocation across the membrane is an intrinsically dynamic process. Molecular dynamics simulations, which can provide atomistic details of molecular motion on ns to ms timescales, are therefore well-suited for studying transport processes. In this review, we outline how molecular dynamics simulations have provided insight into the large scale motions associated with transport of the neurotransmitters, as well as the presence of external and internal gates, the coupling between ion and substrate transport, and differences in the conformational changes induced by substrates and inhibitors.

## Introduction

**FIGURE 1:**
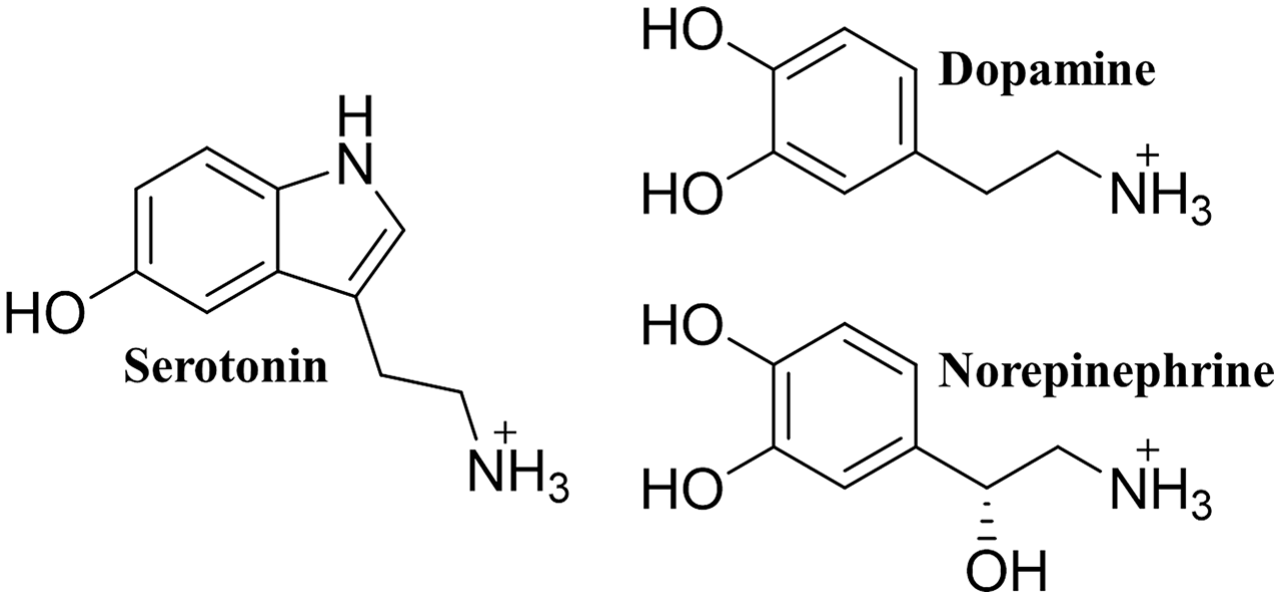
**The monoamine neurotransmitters.** The chemical structure of each of the three monoamine neurotransmitters.

The human monoamine transporters (MATs) are responsible for the reuptake of monoamine neurotransmitters in presynaptic neurons (Giros et al., 1996; Bengel et al., 1998; Xu et al., 2000). There are three different plasma membrane bound MATs, each named according to their main substrate (**Figure 1**), namely the human serotonin transporter (hSERT), the dopamine transporter (hDAT), and the norepinephrine transporter (hNET), all of which utilize the Na^+^ concentration gradient across the membrane to facilitate transport (Gu et al., 1994). Imbalance in neurotransmitter homeostasis is linked to diseases such as major depression, anxiety disorders, attention deficit hyperactivity disorder, schizophrenia, Parkinson’s disease, and obesity (Kristensen et al., 2011), and MATs are thus important pharmaceutical targets. In particular, the treatment of depression has been focused on modulating monoamine neurotransmission, often through inhibition of MATs (Immadisetty et al., 2013). This has resulted in the development of tricyclic antidepressants (TCAs) which target all three MATs as well as selective serotonin reuptake inhibitors (SSRIs) and serotonin and norepinephrine reuptake inhibitors (SNRIs) (Andersen et al., 2009a). Additionally, a number of addictive, illicit drugs, including cocaine and amphetamine, also bind to MATs, and the transporters are thus also considered as potential targets for treating drug addiction (Howell and Kimmel, 2008). During the last decade computational methods have become a prominent tool for investigating MATs in atomic detail. In this review, we will highlight how computational molecular dynamics (MD) studies have been used to generate hypotheses and guide experiments, leading to insight in to how these transporters function with respect to binding of substrates, inhibitors and ions, as well as the intricate details of the mechanism of transport. These insights will not only help guide the development of new drugs with fewer side effects and an improved efficacy, but a greater understanding of the transport process conducted by the MATs will also aid in developing new treatment strategies for less understood diseases, thus improving the lives of the millions of people suffering from mental disorders and obesity world wide.

## Topology and Structure of Mats

To this day, no high-resolution structures of the human MATs are available, and the structural knowledge of human MATs is therefore based on knowledge obtained from other homologous Na^+^-coupled transporters (Manepalli et al., 2012). In this regard, homology models of MATs have yielded unprecedented insight into the structure–function relationship of these transporters by allowing direct observation of possible binding motifs of ligands in the different transporters (Koldsø et al., 2015). These direct observations can then be validated using experimental techniques such as mutational studies and observation of accessibility and dynamics using, e.g., fluorescent probes. Early attempts to model hSERT, hDAT, and hNET were based on crystal structures of distant relatives such as the Na^+^/H^+^ antiporter NhaA and the lactose permease LacY (Ravna et al., 2003a,b; Ravna et al., 2006; Jaroñczyk et al., 2008). However, a major breakthrough in the field occured in 2005 when the first crystal structure of an NSS family member, namely the 1.65 Å structure of the bacterial leucine transporter (LeuT) from *Aquifex aeolicus*, was reported (Yamashita et al., 2005). LeuT has an overall ∼20–25% sequence identity with the human MATs and a ∼50% sequence identity for the residues within a 5 Å radius around the central substrate binding site (Beuming et al., 2006; Koldsø et al., 2013a), making it a reasonable template for modeling the mammalian MATs, in particular with regards to substrate and ligand binding. Although the first crystal structure of LeuT resulted in a quantum leap in the understanding of the structure–function relationship of MATs, the differences between eukaryotic and bacterial transporters still make it difficult to study certain aspects of the MATs. In particular, the eukaryotic members of the NSS family generally have longer C- and N-terminal sections compared to their bacterial counterparts as well as phosphorylation sites and post-translational modifications which are not present in the prokaryotic structures (Pramod et al., 2013). Recently, the first high-resolution structure of a eukaryotic MAT was published in the form of a 2.95 Å resolution crystal structure of the dopamine transporter from *Drosophila melanogaster* (dDAT; Penmatsa et al., 2013), which opens up new possibilities for a deeper understanding of eukaryotic transporters from the NSS family.

**FIGURE 2:**
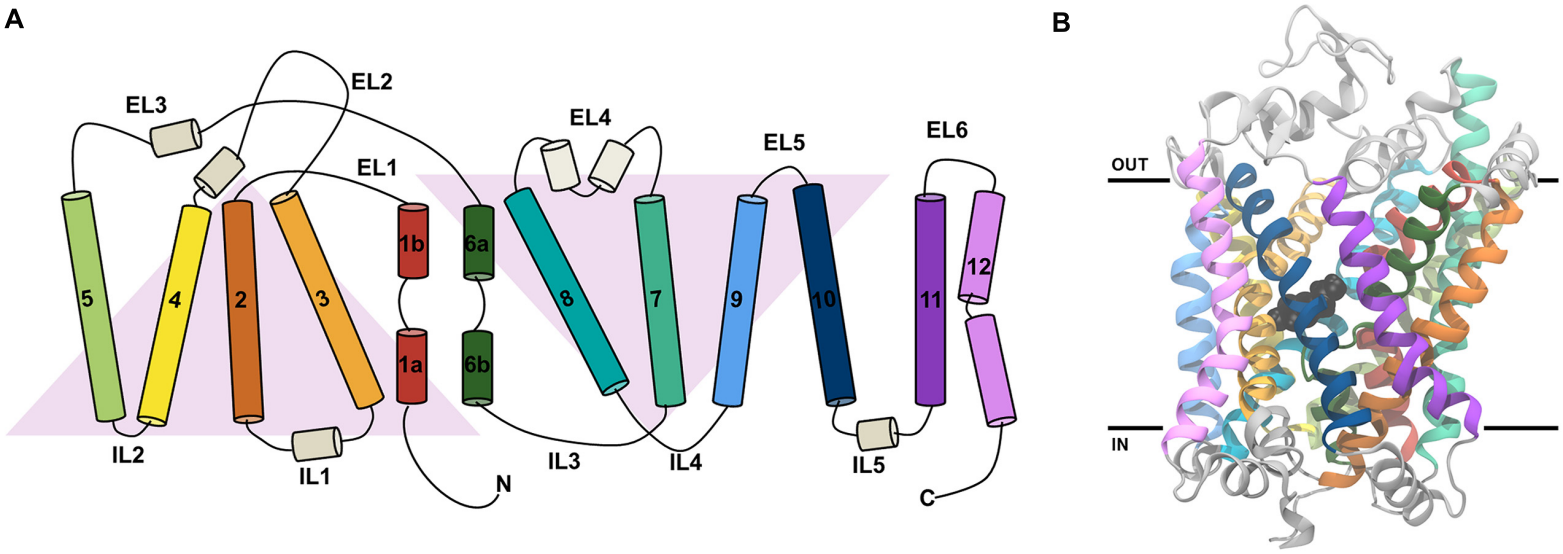
**The topology of the LeuT-fold and the structure of dDAT. (A)** A schematic view of the topology of the 12 TMs in dDAT. TM1-5 and TM6-10 are related by a pseudo twofold rotation as indicated by the gray triangles. **(B)** The structure of dDAT as observed in the X-ray crystal structure with dopamine bound [PDB code 4XP1 (Wang et al., 2015)]. The structure is colored according to the coloring shown in the schematic in part A of this figure. Dopamine in the central binding site is shown in black spheres. The black lines represent the approximate position of the membrane.

**FIGURE 3:**
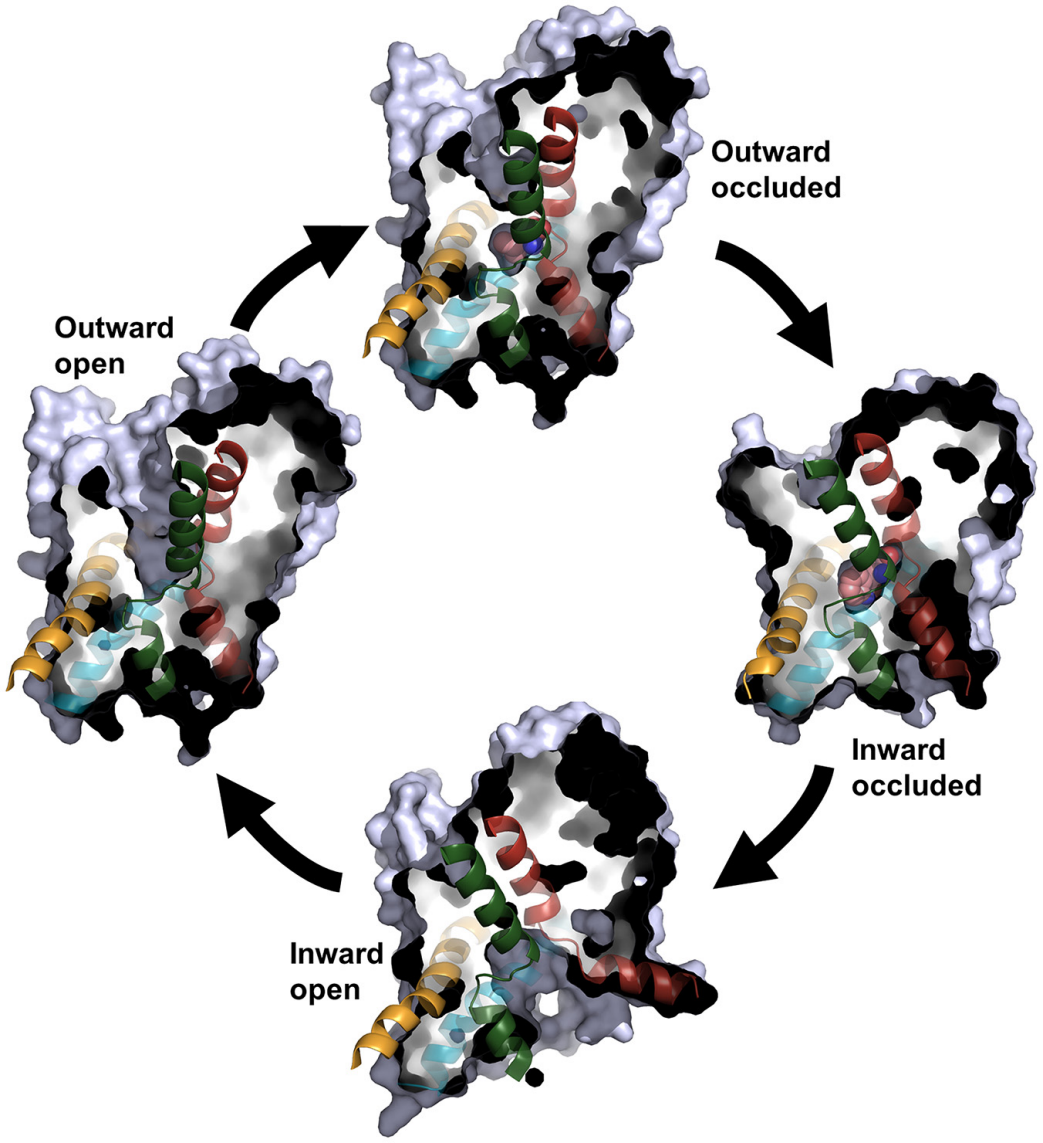
**The transport cycle.** Four of the conformational states in the transport cycle that have been captured in crystal structures of NSS family members are shown as cut-through surfaces. For each state, TM1 and TM6 in the bundle and TM3 and TM8 in the scaffold are shown in red, green, yellow, and blue, respectively. For the outward occluded and inward occluded state, the substrate is shown as spheres with the carbon atoms in pink. The figure is based on the following crystal structures: outward occluded; LeuT, PDB code 2A65 (Yamashita et al., 2005). Inward occluded; MhsT, PDB code 4US3 (Malinauskaite et al., 2014). Inward open; LeuT, PDB code 3TT3 (Krishnamurthy and Gouaux, 2012). Outward open; LeuT, PDB code 3TT1 (Krishnamurthy and Gouaux, 2012).

Both the LeuT and dDAT structures contain intracellular N- and C-termini, 12 transmembrane helices (TMs) named TM1-12 (**Figures 2A,B**), as well as six extracellular and five intracellular loops (EL1-6 and IL1-5). The first ten TMs are arranged in a pseudo-symmetric fold, known as the LeuT-fold, in which TM1-5 has the same overall internal arrangement as TM6-10 (Yamashita et al., 2005; Forrest et al., 2008). The primary substrate binding site is located in the center of the transporter and is formed by TM1, TM3, TM6, and TM8. The transport mechanism of secondary active transporters, such as those in the NSS family, is thought to follow the alternating access mechanism (Jardetzky, 1966), entailing that the transporter alternates between conformational states, in which the substrate binding site is accessible either from one side of the membrane or the other. LeuT has been crystallized in both outward- and inward-facing states (Yamashita et al., 2005; Singh et al., 2008; Krishnamurthy and Gouaux, 2012), whereas crystal structures of dDAT capture the transporter in an outward-open or outward-occluded state (Penmatsa et al., 2013; Wang et al., 2015) (**Figure 3**). Based on the crystal structure of the outward-facing conformation of LeuT, and the observed pseudo-symmetry of the LeuT-fold, Forrest et al. (2008) proposed a structure of the inward-facing conformation of LeuT (Forrest et al., 2008) prior to the capture of this state in LeuT crystal structures. A comparison of the outward- and inward-facing conformation then led to the formulation of the rocking bundle transport mechanism (Forrest and Rudnick, 2009). According to this mechanism four of the TMs, two from each inverted repeat (TM1, TM2, TM6, TM7), form a bundle which is partly surrounded by six helices, three from each inverted repeat (TM3-5, TM8-10), which acts as a stationary scaffold. The bundle helices are hypothesized to rock back and forth resulting in the central binding site alternating between being accessible to the extra- or intracellular environment (**Figure 3**). Additionally, a two-substrate mechanism has been proposed for LeuT-fold transporters (Shi et al., 2008; Shan et al., 2011). This mechanism entails that binding of a second substrate in a binding site in the extracellular vestibule is necessary for triggering the release of substrate from the primary binding site to the intracellular milieu.

## Substrate, Ion, And Inhibitor Binding

### The Primary Substrate Binding Site

**FIGURE 4:**
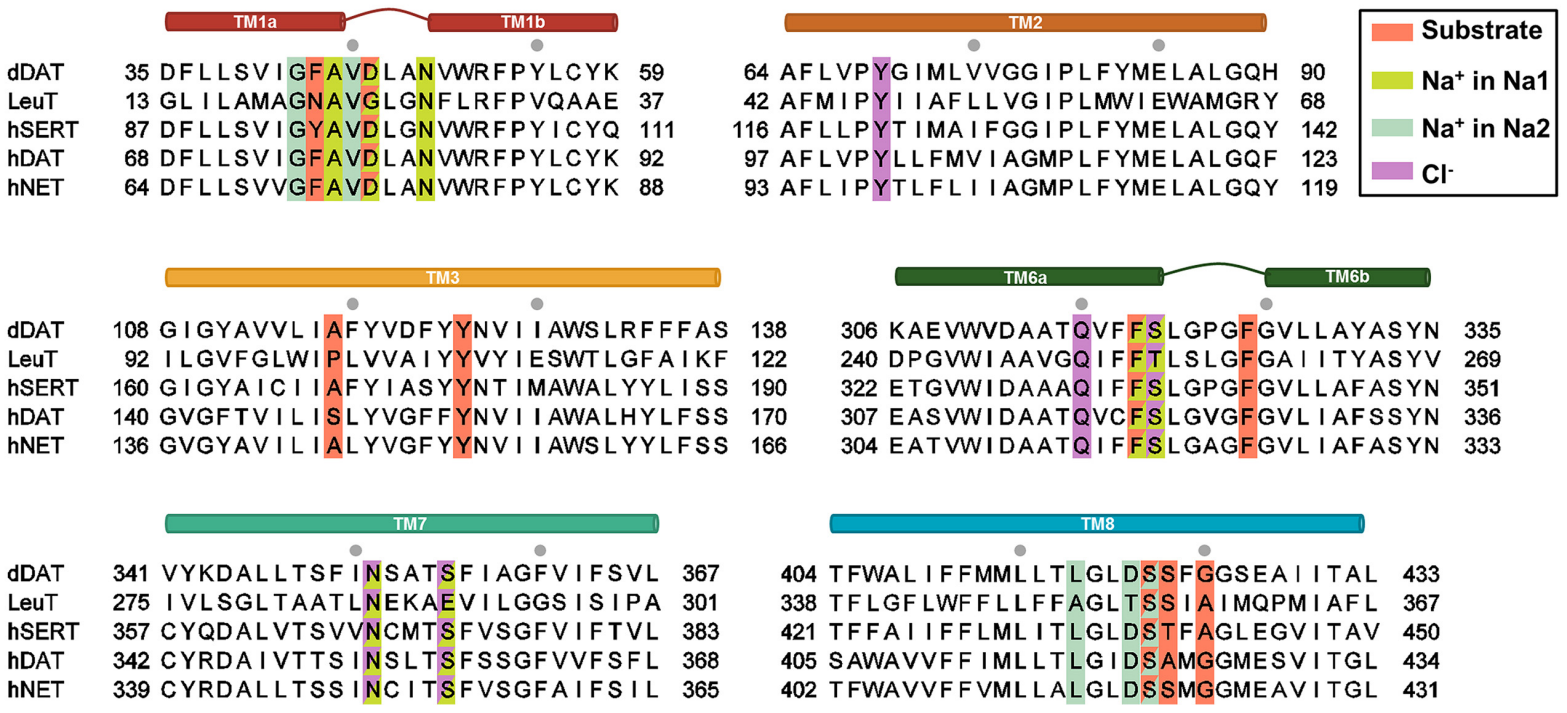
**Sequence alignment of the regions involved in substrate and ion binding.** The alignment has been made using ClustalO. Residues proposed by MD studies to interact with either substrates or ions are colored accordingly. Residues interacting with more than one substrate/ion are displayed in two colors. The gray dots represent intervals of ten residues.

The binding interactions of the endogenous substrates have been thoroughly examined using computational methods. All three substrates share similar primary binding interactions, and in fact the substrates can also to some extent be transported by some of the other transporters (Gu et al., 1994; Zhou et al., 2005; Larsen et al., 2011). The substrates are believed to carry a positive charge at their primary amine when they are transported (Keyes and Rudnick, 1982; Berfield et al., 1999) and are thus capable of forming a salt bridge with a negatively charged aspartate present in the binding site (Barker et al., 1999). In all three binding sites the majority of residues are hydrophobic (**Figure 4**); however, a few polar residues are able to form stronger interactions with the substrates (Koldsø et al., 2013a), a feature which can be exploited when designing selective ligands.

**FIGURE 5:**
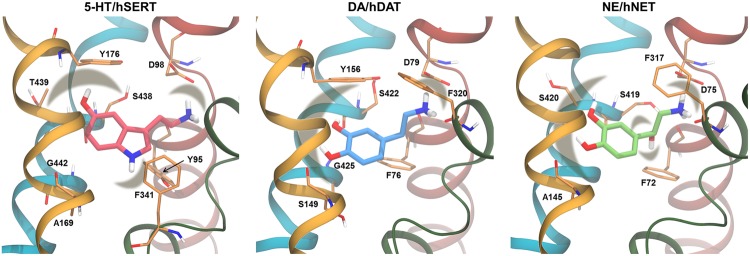
**Substrate binding sites.** The residues proposed to interact with the substrate based on MD studies are shown as thin sticks with the carbon atoms in orange. In cartoon is shown TM1 (red), TM3 (yellow), TM 6 (green), and TM8 (blue). The substrates are shown in thicker sticks with the carbon atoms in pink for 5-HT, blue for DA, and green for NE. The shaded areas mark interactions between the substrate and the binding site residues. Models of hSERT, hDAT, and hNET are from (Koldsø et al., 2013a).

For hSERT, several molecular docking and MD simulation studies have found that the primary amine in the substrate serotonin (5-HT) forms a salt bridge with D98 (Jørgensen et al., 2007; Celik et al., 2008; Gabrielsen et al., 2012), as seen in **Figure 5**. The amine in the indole moiety of 5-HT and the hydroxyl group are expected to participate in hydrogen bonds with residues in the transporter binding site; however, multiple different interaction partners have been suggested based on modeling. Jørgensen et al. (2007) found that the secondary amine interacts with T439 and the hydroxyl group interacts with G442. Gabrielsen et al. (2012) found two possible binding modes of 5-HT; one, termed mode A, in which the secondary amine is located between Y95 and F341 and the hydroxyl group is pointing toward Y176, S438, and T439, and another one, termed mode B, in which the indole ring is flipped such that the secondary amine points toward Y176 and S438 and the hydroxyl group is in the vicinity of A169 and F341 (Gabrielsen et al., 2012). The latter binding mode is similar to the one described by Jørgensen et al. (2007). Gabrielsen et al. (2012) performed MD simulations of hSERT in complex with 5-HT in each of the two binding modes, and found that only 5-HT binding in mode B is able to induce conformational changes in hSERT, and based on this, mode B was suggested as the most likely binding mode. However, Celik et al. (2008) found, based on docking studies and an exhaustive structure–activity relationship study using the paired mutant-ligand analog complementation approach, that 5-HT binds in mode A (Celik et al., 2008). This experimentally validated binding mode has been used in an unbiased MD study of hSERT in which a substrate-induced conformational change from outward- to inward-facing was observed (Koldsø et al., 2011), which further corroborates that mode A is the most likely binding mode for 5-HT.

Similar studies have been performed for hDAT, focusing both on the primary site and a vestibular site, located ∼10 Å above the primary binding site. Most homology models have been constructed based on LeuT template structures, but also the Na^+^/H^+^ antiporter from *Escherichia coli* (NhaA) (Ravna et al., 2003b) has been used. Since NhaA has a different fold than LeuT (Forrest et al., 2011), the models based on NhaA are very different from the LeuT-based models and the dDAT crystal structure, and will thus not be included in the following. Common for the studies focusing on the primary binding site is the observation of a salt bridge between the primary amine of the substrate dopamine (DA) and D79, however, the amine is also likely interacting with the backbone of F76 (Huang and Zhan, 2007), S422 (Huang and Zhan, 2007), or F320 (Koldsø et al., 2013a) through hydrogen bonds. The *para*-hydroxyl group is observed to interact with S149 (Koldsø et al., 2013a) or the backbone of G425 or S422 (Huang and Zhan, 2007) (**Figure 5**). Studies of a homology model of the rat DAT (rDAT) showed similarly that the charged amine of DA interacts with either D79 (corresponding to D79 in hDAT) or the backbone of F76 (F76 in hDAT) while the *para*-hydroxyl group interacts with S421 (S422 in hDAT) (Merchant and Madura, 2012). Huang and Zhan (2007) observed π–π interactions between DA and Y156, and cation-π interactions between the primary amine of DA and the aromatic ring system of F76, which has not been reported by other groups. Two different binding modes of DA were found by Koldsø et al. (2013a) using docking methods where one binding mode was an 180° rotation around the long axis of DA compared to the other binding mode. The two binding modes were distinguishable by the interaction of the *meta*-hydroxyl group which was either with S149 or the backbone of S422. In MD simulations, DA was found to fluctuate between the two binding modes (Koldsø et al., 2013a). Thus, it is likely that DA binding to hDAT is rather flexible and involves shifts between different interaction partners. Shan et al. (2011) reported a binding pattern similar to the one reported by Huang and Zhan (2007) when performing steered MD (SMD) simulations of DA being pulled from the primary binding site in hDAT toward the extracellular milieu.

Very few MD simulation studies have been performed for hNET. Using both docking and MD simulations Koldsø et al. (2013a) found that the positively charged amine of norepinephrine (NE) forms a salt bridge interaction with D75 or a hydrogen bond to F317 similar to the other monoamines in their respective transporters. Additionally, the *meta*-hydroxyl group was found to interact with S420 while the *para*-hydroxyl group interacts with the backbone of A145. The hydroxyl group on the alkyl chain of NE was found to interact with the backbone of either S419 or F72.

It is evident that it is possible to predict binding modes of substrates using molecular mechanical approaches and to validate them by use of MD simulations and experimental methods as demonstrated above. Binding of substrates to MATs appear to have several features in common such as salt bridge formation to an aspartate in TM1, hydrogen bonding to polar residues in TM3 and TM8, and hydrophobic interactions with conserved residues in the binding site as illustrated in **Figures 4** and **5**.

### Ion Binding Sites

Due to the high concentrations of monoamine neurotransmitters inside neurons, a source of energy is necessary for facilitating the transport of the monoamine substrates across the membrane against their concentration gradient. For all three MATs, transport of substrate is coupled to co-transport of Na^+^ and Cl^-^ ions along their concentration gradient (Gu et al., 1994), while hSERT also requires counter-transport of K^+^ (Nelson and Rudnick, 1979). The transport stoichiometry is believed to be 1:1:1:1 (hSERT), 1:1:1:0 (hNET), and 1:2:1:0 (hDAT) for substrate/Na^+^/Cl^-^/K^+^ (Talvenheimo et al., 1983; Gu et al., 1994, 1996). Until now, it is only the two Na^+^ ion binding sites, known as Na1 and Na2, and the Cl^-^ ion binding site that have been identified through X-ray crystallography of LeuT (Yamashita et al., 2005) and dDAT (Penmatsa et al., 2013), while the ion binding site for K^+^ in hSERT remains unidentified. The observed coordination geometry in the Na^+^ ion binding sites changes between the structures of LeuT and dDAT, suggesting that the coordination geometry of the Na^+^ ions might have subtle differences between the MATs.

**FIGURE 6:**
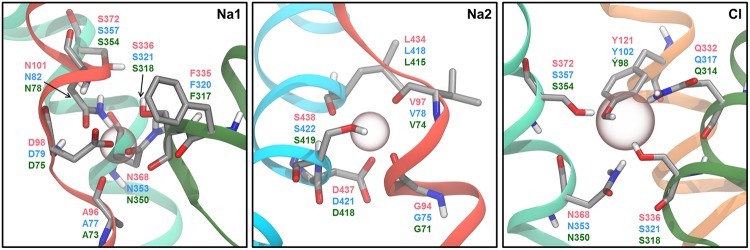
**Ion binding sites.** Residues proprosed by MD studies to be involved in ion binding are shown as sticks with the carbon atoms in gray. The residue indices are given in red for hSERT, blue for hDAT, and green for hNET. The approximate position of each ion is indicated by a sphere. The figure is based on a homology model of hSERT (Koldsø et al., 2013a).

Although only a single Na^+^ ion is expected to be transported along with 5-HT in hSERT, it is commonly believed that two Na^+^ ions bind to both the Na1 and the Na2 site in hSERT simultaneously. Early, 17 ns long MD simulations of a hSERT homology model based on LeuT suggested pentacoordination of the ion in the Na1 site by the sidechain of residues D98, N101, and S336 and the backbone carbonyl of A96 and F335 (**Figure 6**; Jørgensen et al., 2007). However, in several more recent simulation studies, the Na^+^ ion in the Na1 site has been observed to be stably hexacoordinated by the sidechain of D98, N368, and N101, the backbone carbonyl of A96 as well as both the sidechain hydroxyl and backbone carbonyl of S336 (Henry et al., 2011; Koldsø et al., 2013a; Felts et al., 2014), all of which correspond to the coordinating residues in the Na1 site of LeuT. For the Na2 site, pentacoordination involving the backbone carbonyls of G94 and V97, both sidechain oxygen atoms of D437, and either the hydroxyl group of S438 (Felts et al., 2014) or the backbone carbonyl of L434 (Jørgensen et al., 2007) has been suggested. Additionally, hexacoordination, where both S438 and L434 participate in the coordination, has also been proposed (Celik et al., 2008; Koldsø et al., 2011). Since LeuT is a Cl^-^-independent transporter, the transporter does not contain a Cl^-^ ion binding site; however, it has been shown that the side chain of the residue E290 in LeuT overlaps with the position of the Cl^-^ ion binding site in the human neurotransmitter transporters (Forrest et al., 2007; Zomot et al., 2007). Based on this, a number of different coordinating residues and coordinating geometries have been proposed from MD studies of LeuT-based hSERT homology models. In the dDAT structures (Penmatsa et al., 2013, 2015; Wang et al., 2015), the bound Cl^-^ ion is coordinated in a tetrahedral fashion, and it is likely that the corresponding residues in hSERT, Y121, Q332, S336, and S372, coordinate the Cl^-^ ion, considering that these four residues are all conserved among the human MATs and dDAT. In addition, N368 has also been proposed to be involved in Cl^-^ coordination by several studies (Celik et al., 2008; Henry et al., 2011; Koldsø et al., 2011; Felts et al., 2014). Interestingly, it has been found that mutating N101 in the Na1 site of hSERT to either alanine or cysteine gives rise to Cl^-^-independent substrate transport (Henry et al., 2011; Felts et al., 2014). In MD simulations of wild type hSERT with and without Cl^-^ bound, it has been observed that lack of Cl^-^ causes D98 to coordinate only the Na^+^ ion in the Na1 site in a bidentate fashion rather than interacting both with the Na^+^ ion and the charged amine group of 5-HT (Henry et al., 2011; Felts et al., 2014). On the other hand, simulations of the N101A mutant with an empty Cl^-^ site display interaction from D98 to both the Na^+^ ion and the substrate, which may explain the lack of Cl^-^ dependence for this mutant. In these simulations it was also observed that the positioning of S336 and N368, both of which have been suggested to interact simultaneously with the Cl^-^ ion and the Na^+^ ion in the Na1 site, play an important role in ion-coupled substrate movement. Additionally, the N101A mutation also allows Ca^2+^ to substitute for Na^+^ in terms of facilitating transport. Based on a combination of biochemical experiments and MD simulations, it has been suggested that Ca^2+^ binds in the Na1 site of the N101A mutant, and is not transported along with substrate (Felts et al., 2014). The results could indicate that it is only the Na^+^ ion in the Na2 site in wild type hSERT that is co-transported with the substrate and that the role of the Na^+^ ion the Na1 site is mainly related to substrate binding.

In the case of hDAT, MD simulations performed by Huang and Zhan (2007) show that the coordination of the Na^+^ ions shifts between penta- and hexacoordination, and that the ratio between these two coordination geometries change upon DA binding in the primary binding site. Furthermore, they found that the coordinating amino acids also differed when performing simulations of hDAT with and without DA bound. Specifically, they find that the ion in the Na1 site is coordinated by A77, N82, S321, F320, and D79 when DA is occupying the primary binding site, but when the primary binding site is empty the ion in the Na1 site is primarily hexacoordinated by A77, N82, S321, N353, S357, and F76, thus changing the coordinating state of the ion as well as several of the coordinating residues (Huang and Zhan, 2007). However, it should be noted that the simulations were performed without Cl^-^ bound to hDAT, which is likely to have affected the results. For instance, S357 is expected to coordinate Cl^-^ (Penmatsa et al., 2013), and would not be able to coordinate both Na^+^ and Cl^-^ simultaneously. Koldsø et al. (2013a) also found that the ion in the Na1 site is pentacoordinated in a DA-occupied hDAT model; however, they found that Na^+^ interacts with N353 and not F320 as Huang and Zhan observed. For the ion in the Na2 site, Huang and Zhan (2007) also observed a change in coordination state caused by the presence of substrate. When simulations of hDAT were performed in the absence of DA within the primary binding site, the ion in the Na2 site is hexacoordinated by G75, D79, L418, the hydroxyl group of S422 and both sidechain oxygen atoms of D421 most of the simulation time. However, when DA is bound, pentacoordination by G75, V78, L418, and both sidechain oxygen atoms of D421 is observed in the majority of the simulation time. When the ion in the Na2 site shifts to pentacoordination in simulations of hDAT without DA it is the coordination by S422 that is lost (Huang and Zhan, 2007). Thus the pentacoordination of the ion in the Na2 site is not the same in simulations of hDAT with and without DA, as V78 is exchanged for D79 in simulations of hDAT with DA occupying the primary binding site. Koldsø et al. (2013a) have reported the ion in the Na2 site to be coordinated by the same residues as Huang and Zhan (2007) found for DA bound hDAT, but they only observed an interaction with one of the side chain oxygen atoms of D421 corresponding to pentacoordination (Koldsø et al., 2013a) and not hexacoordination as Huang and Zhan (2007) reported to be most common. A study by Shan et al. (2011) found that the coordination of the ion in the Na2 site by L418 was abolished when a DA molecule was placed in both the primary binding site and the vestibular site of hDAT using SMD. They found that the change in coordination was due to a rotation of L418 which makes the sidechain of L418 able to interact with W84 instead of the Na^+^ ion. Furthermore, they observe solvation of the Na2 site from the intracellular side after this change in coordination, suggesting this change in coordination might be necessary for inward release of Na^+^.

The dynamics of the Cl^-^ site in hDAT has to our knowledge not yet been reported by anyone. However, Koldsø and co-workers report a tetrahedral coordination of Cl^-^ to Y102, S321, N353, and S357 in their homology model.

The stabilization of an outward-facing conformation of hDAT by Zn^2+^ has been long established (Richfield, 1993), but the interactions allowing for this effect are less well-known. A homology model of hDAT has been constructed based on LeuT for the outward-facing conformation, where the Zn^2+^ binding site has been used as a restraint in the model making process (Stockner et al., 2013). This was shown to change the observed behavior of hDAT in MD simulations, compared to MD simulations of homology models constructed without focus on these possible restraints. Based on MD simulations of this homology model, it was found that the Zn^2+^ site not only consisted of H193, H375, and E396 as determined by other methods, but also included coordination to D206. Furthermore, the flexibility of EL2 was decreased in this homology model, and it was observed that the Zn^2+^ site was broken when the bundle domain of hDAT rotated as part of the conformational change needed to shift to the inward-facing conformation of hDAT. This site disruption was found by combining the new EL2 loop conformation with a structure of LeuT in the inward-facing conformation (Stockner et al., 2013).

The dynamics of the coordination of the bound Na^+^ ions and Cl^-^ ion in hNET have not been reported to our knowledge. Koldsø et al. (2013a) reports the coordination of the ions in a homology model that has been energy minimized. They find that the ion in the Na1 site is pentacoordinated by A73, D75, N78, S318, and N350, while the ion in the Na2 site is pentacoordinated by G71, V74, L415, D418, and S419. Similar to what has been suggested for hSERT and hDAT, they observe Cl^-^ to be tetracoordinated by Y98, S318, N350, and S354.

Several coordination geometries and coordinating residues in the Na^+^ and Cl^-^ sites have been proposed based on homology modeling and MD simulations as illustrated above. Despite Na^+^ ion coordination changing between penta- and hexacoordination, a common motif of binding appears for all ion sites (**Figures 4** and **5**), suggesting that the overall coordination of ions in MATs is similar although not identical. As described above, it has been possible to observe changes in coordination geometry of ion sites depending on substrate binding, and to predict changes in binding site behavior and ion selectivity using MD simulations. This has led to a greater understanding of the intricate details governing ion dependent transport.

### Inhibitor Binding

**FIGURE 7:**
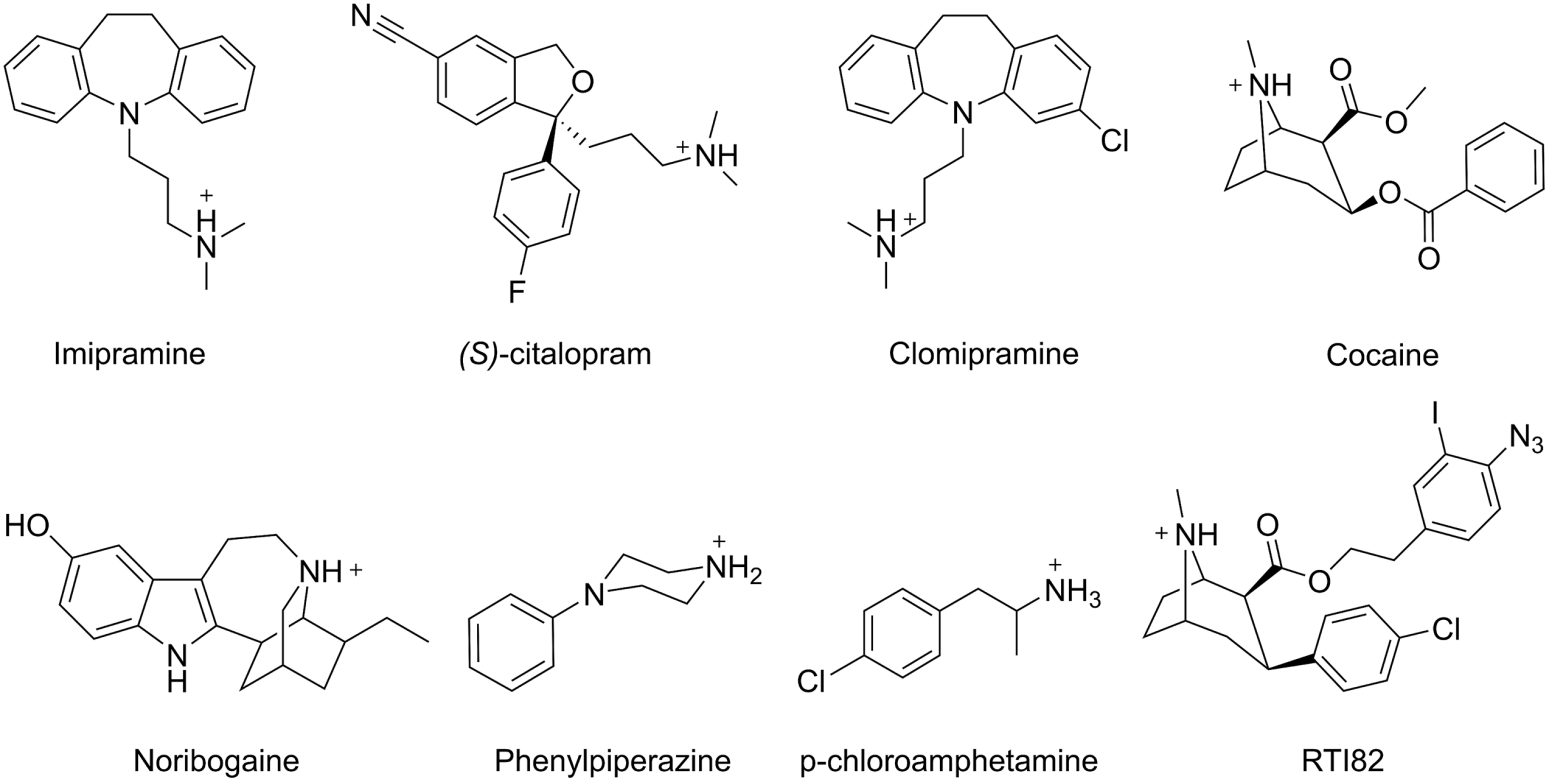
**Chemical structures of selected MAT inhibitors**.

The most commonly used antidepressants, such as TCAs, SSRIs, and SNRIs, all function by inhibiting the MATs, thereby increasing the monoamine concentration in the synaptic cleft (Immadisetty et al., 2013). Due to their clinical importance, there is a great interest in understanding the structural basis underlying their action. Although crystal structures of LeuT with either an SSRI or a TCA bound in the extracellular vestibule have been published (Singh et al., 2007; Zhou et al., 2007, 2009), evidence from both experimental and computational studies of the human MATs points toward the inhibitors primarily binding in the central substrate binding site (Andersen et al., 2009b, 2010, 2011, 2014; Koldsø et al., 2010; Sinning et al., 2010). In accordance with this, MD simulations of hSERT with the TCA imipramine (**Figure 7**) bound in either the central binding site or the extracellular vestibular binding site have suggested that imipramine binds stably in the primary site and unstably in the extracellular vestibule (Sinning et al., 2010). The result is supported by dDAT crystal structures which include several types of antidepressants bound in the primary site (Penmatsa et al., 2013, 2015; Wang et al., 2015), and by the fact that crystal structures of LeuT-variants, in which the primary site has been made MAT-like, also display binding of TCAs, SSRIs, and SNRIs in the primary site (Wang et al., 2013). On the other hand, it is known that hSERT contains an additional low-affinity allosteric site, and that binding of ligands in this site affects the dissociation rate of ligands from the central binding site (Plenge and Mellerup, 1997). Using a combination of docking, MD simulations, and mutagenesis, Plenge et al. (2012) showed that the allosteric site is positioned in the extracellular vestibule. An iterative approach was used to find the optimal binding mode of the SSRI *(S)-*citalopram and the TCA clomipramine (**Figure 7**) in the extracellular vestibule of hSERT with *(S)-*citalopram simultaneously bound in the central binding site. First, an induced fit docking was performed, followed by an MD simulation of the top-ranked pose. If unstable binding was observed during the simulation, the ligand was re-docked into the MD-altered binding site, and a new simulation was performed. This was repeated until a stable binding mode was obtained, such that residues important for binding could be proposed (Plenge et al., 2012). Based on the results, a number of residues in the extracellular vestibule were mutated and the majority caused a change in the potency of *(S)*-citalopram and clomipramine in inhibiting the dissociation of *(S)*-citalopram from the high-affinity central binding site, which validates both the position of the allosteric site and the proposed binding modes of the two inhibitors in the allosteric site.

Besides pharmaceutical drugs, MATs are also the targets of drugs of abuse such as cocaine, amphetamine and 3,4-methylenedioxy-methamphetamine (commonly known as ‘ecstasy’) (Howell and Kimmel, 2008). Among the MATs, the primary target of cocaine is hDAT, and there are multiple MD studies aiming at elucidating its mechanism of inhibition. The location of the binding site of cocaine in hDAT has been debated, and several different computational as well as experimental methods have been employed in an attempt to determine if cocaine binds to the primary binding site or the vestibular binding site. Merchant and Madura (2012) used multi-configuration thermodynamic integration (MCTI) to determine the free energy change needed for a cocaine molecule to pass through rDAT. The resulting free energy profile showed an energetic minimum for cocaine in the vestibular site, but not in the primary binding site. No direct binding interactions between cocaine and the primary binding site were observed, and they report cocaine as being too large to fit in the binding site. Interestingly, they do not report any specific interactions between cocaine and rDAT when in the vestibular site either, but this site is proposed to be able to expand to allow cocaine binding (Merchant and Madura, 2012). Huang et al. (2009) investigated cocaine binding to the vestibular binding site in hDAT while the primary binding site was occupied by DA. They found cocaine to be able to bind stably throughout 4.5 ns of MD simulation (Huang et al., 2009), and observed the positively charged amine of cocaine to be close to Y88, although a direct cation-π interaction was not seen. Cocaine binding was also found to be stabilized by interactions with L80, A81, I159, F155, Y156, and F320 in the vestibular binding site. Based on this result, it was suggested that DA binds first, followed by cocaine binding in the vestibular site, thus trapping DA in the primary site while not allowing hDAT to continue the conformational changes needed to release DA to the cytoplasm. Dahal et al. (2014) attempted to determine the binding site of cocaine by performing docking and MD simulations of rDAT with a cocaine analog, RTI82, bound. They found RTI82 to bind in the primary binding site with the charged amine stably interacting with D79 (D79 in hDAT), and the chloro-substituent close to N157 (N157 in hDAT) throughout 60 ns of MD simulations. The azidoiodophenyl group is pointing toward the vestibular site, with the azido group interacting with F319 (F320 in hDAT). Dahal et al. (2014) also performed biochemical experiments utilizing the ability of RTI82 to covalently attach to –CH and –NH groups in hDAT upon photoactivation, and found that RTI82 is able to covalently bind to F320 in hDAT further supporting their binding hypothesis. They suggest that the observed binding pattern for RTI82 can be transferred to cocaine binding in the primary binding site (Dahal et al., 2014). However, it should be noted that RTI82 is a significantly larger molecule than cocaine (**Figure 7**), which could suggest that there may be some differences in the interaction pattern for the two compounds. Taken together, MD simulations mainly indicate that cocaine binds to the vestibular site in hDAT and rDAT; however, the recent dDAT crystal structure shows cocaine co-crystalized in the primary binding site (Wang et al., 2015), suggesting that species-dependent differences in the mechanism of inhibition by cocaine could perhaps exists. Alternatively, the results could indicate that studies based on hDAT in the outward occluded state, rather than the outward open state, are not valid in the context of cocaine binding predictions since cocaine is too big to bind in this conformation thus leading to false predictions. Cocaine binding to hSERT has also been studied using docking and MD simulations; Koldsø et al. (2013b) showed that cocaine positioned in the central binding site primarily stabilized an outward-facing conformation during the simulations, in accordance with what is expected based on accesability measurements (Zhang and Rudnick, 2006). Similarly, it was also observed that positioning of noribogaine, a non-competitive inhibitor of hSERT that traps the transporter in an inward-facing conformation (Jacobs et al., 2007), in the primary binding site caused an opening of the transporter to the intracellular side. These results imply that MD simulations can be applied to observe inhibitor-induced conformational changes that are consistent with experimental results and furthermore substantiates that both noribogaine and cocaine bind in the primary binding site of hSERT.

While many of the MAT inhibitors, such as SSRIs and cocaine, competitively inhibit substrate transport, amphetamines function as exogenous substrates and are believed to be transported by the MATs from the synaptic cleft to the intracellular space. Additionally, amphetamines induce substrate eﬄux facilitated by the MATs, which leads to an increase in the cytosolic monoamine concentration (Sitte and Freissmuth, 2015). The role of the N-terminus of MATs in amphetamine action has been studied by Sucic et al. (2010) using a combination of MD simulations and biochemical experiments. In this study, it was shown by mutational experiments that changing T81 in the N-terminus of hSERT to alanine abolishes *para*-chloroamphetamine induced eﬄux of substrate. T81 is part of a stretch of residues that are conserved among the MATs, and the corresponding mutations in hDAT and hNET led to similar results. Short MD simulations of wild type hSERT (3 ns) as well as the *in silico* T81A mutant (6 ns), using a homology model in which the first 78 residues have been truncated in both setups, showed that the hydroxyl group of T81 forms a hydrogen bond with the backbone carbonyl of Y350, which is absent for the T81A mutant. Additionally, the simulations showed that when the T81A mutation is introduced, the cytoplasmic end of TM1 moves away from TM6, IL3, and IL2, leading to a more inward-facing conformation. Furthermore, the distance between the N- and C-terminus is increased in this mutant during 6 ns of MD simulation (Sucic et al., 2010). Thus, based on the simulations it could be predicted that ibogaine, which stabilizes the inward-facing conformation, should have a higher affinity for the T81A mutant, while imipramine, which stabilizes the outward-facing conformation should have a lower affinity. Subsequent experiments using the T81A mutant showed that the changes in binding affinity for imipramine and noribogaine predicted by MD simulations could indeed be verified (Sucic et al., 2010). Additionally, fluorescence resonance energy transfer experiments showed an increase in the distance between the N- and C-terminus for the T81A mutant compared to wild-type hSERT, which is also in agreement with the results from the MD simulations. Interestingly, expression of a hSERT variant with the first 64 residues deleted showed that omission of the N-terminal residues has similar effects on the *para*-chloroamphetamine induced eﬄux as the T81A mutant. Overall, the results suggest that the N-terminus plays a direct role in driving the transporter into a state that supports eﬄux induced by amphetamines. In an effort to decipher the mechanism behind this, Khelashvili et al. (2015) constructed a model of the N-terminus of hDAT and performed MD simulations of only the N-terminus anchored to a mixed lipid membrane. It was found that the terminal was structured in such away that several lysine residues clustered together and stably interacted with the negatively charged phosphatidylinositol 4,5-biphosphate lipids in the inner leaflet of the membrane throughout more than 550 ns simulations. Simulations of the N-terminal with serine to aspartate mutations that imitate phosphorylation showed a disruption of the N-terminal anchoring in the membrane suggesting that the anchored conformation is necessary for amphetamine-induced eﬄux since phosphorylation of the N-terminal has been shown to regulate eﬄux (Khelashvili et al., 2015). Phenylpiperazine (PP) also acts as monoamine releaser. The binding of PP and PP analogs to hSERT and hDAT has been studied by a combination of docking, MD simulations and biochemical experiments, and it was found that this class of compounds binds in the primary binding pocket of the transporters (Severinsen et al., 2012). Additionally it was found that both PP and the PP analogs formed stable interactions with D98 within hSERT during MD simulations. A PP analog, 3-hydroxyl PP, was found to bind in two different orientations differing by a 180° rotation. Both of these binding modes remained stable in terms of the hydrogen bonding pattern observed during MD simulations. On the other hand, the phenyl and the piperazine rings were observed to rotate with respect to each other in simulations of the unsubstituated PP while maintaining the saltbridge between the positively charged amine group of the ligand and D98 in hSERT (Severinsen et al., 2012).

As demonstrated above, it is possible to observe inhibitor-induced conformational changes during unbiased MD simulations despite the computational limitations in simulation time. In addition, the different mechanisms of inhibition by several classes of inhibitors can be distinguished in MD simulations allowing great insight into the mechanistic details of MAT inhibition.

## The Transport Mechanism

In spite of progress in determining how neurotransmitters are transported across the membrane, the molecular mechanism is still not well-understood. Several conformational states have been captured in crystal structures of LeuT (Yamashita et al., 2005; Singh et al., 2008; Krishnamurthy and Gouaux, 2012), dDAT (Penmatsa et al., 2013, 2015; Wang et al., 2015), and in crystal structures of LeuT engineered to resemble the central binding sites in MATs (Wang et al., 2013). However, each structure only represents a single snapshot in the transport cycle, and the transition from one state to another cannot readibly be inferred. However, MD simulations are excellent for obtaining knowledge on dynamic processes such as possible pathways between two conformational states. Both unbiased equilibrium simulations and simulations biased toward observing specific events have proven to be very useful in deciphering the mechanism of action of MATs.

### The Role of Substrate Binding in the Vestibular Site

**FIGURE 8:**
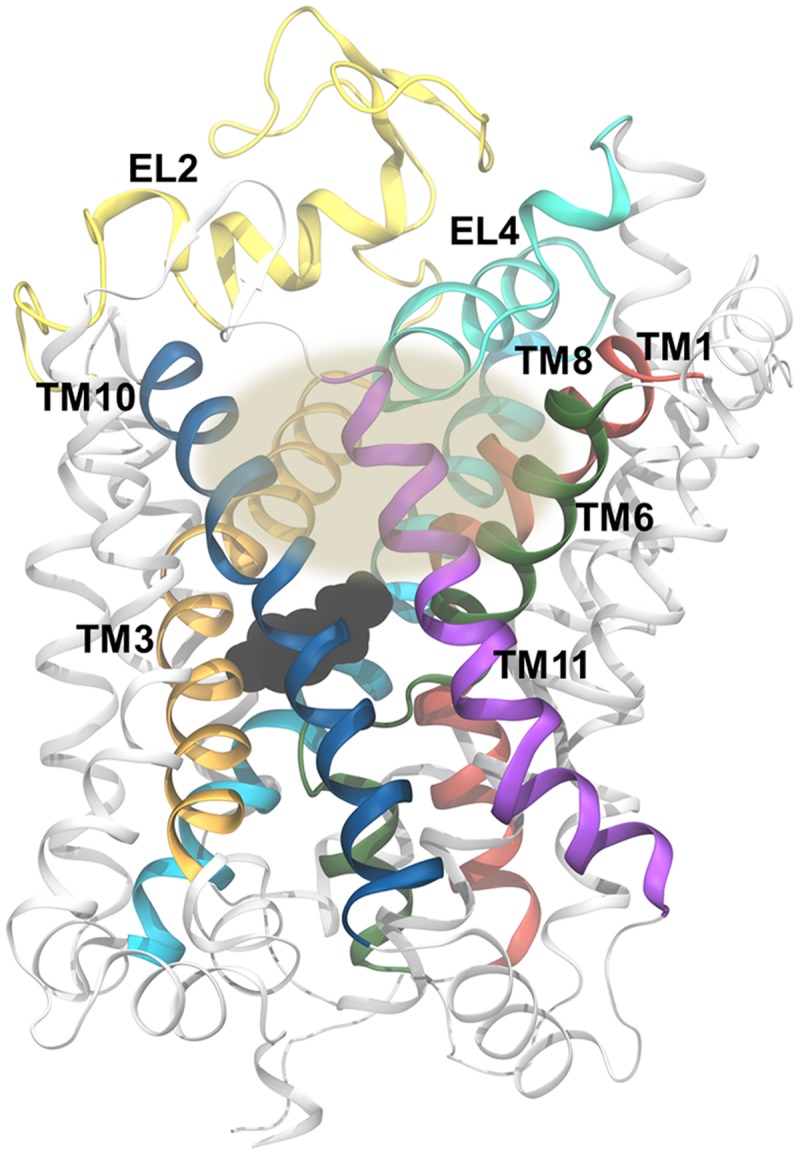
**The vestibular binding site.** The gray oval marks the approximate position of the vestbular binding site, which is shown here for dDAT [PDB code 4XP1 (Wang et al., 2015)]. The site is flanked by TM1 (red), TM3 (orange), TM6 (green), TM8 (cyan), TM10 (blue), and EL4 (aquamarine). EL2 is shown in yellow and the remaining part of the transporter is shown in white. Dopamine, bound in the central binding site, is shown in black spheres.

It has been debated whether substrate binds only in the primary binding site of LeuT and MATs (Reyes and Tavoulari, 2011) or if binding of a second substrate in the extracellular vestibule (**Figure 8**) is needed to trigger substrate release from the primary binding site as has been proposed for LeuT (Shi et al., 2008) and hDAT (Shan et al., 2011). Several computational methods have been applied to try to determine this. Merchant and Madura, (2012) calculated a free energy profile of DA moving through rDAT using the MCTI method, and found that DA would pass through an energetic minimum in both the primary and the vestibular binding site (Merchant and Madura, 2012). However, when they applied random accelerated MD (RAMD) to assess the possible release pathways of DA from the primary binding site, they did not find a clear release pathway to the intracellular side when DA was occupying both binding sites, but they did observe a clear pathway when performing simulations of rDAT with DA bound only in the primary binding site. They suggest that only one DA molecule is needed during transport, and that the energetic minimum found in the vestibular binding site is responsible for moving DA from the extracellular milieu to the primary binding site, thus serving as a stepping stone between the solvent and the binding site. An SMD study by Shan et al. (2011) found that the release of DA to the cytoplasm was improved by having both the primary and the vestibular binding site occupied by DA due to an increase in interactions between DA in the primary site and water molecules from the cytoplasm when DA is also occupying the vestibular site. Due to a movement of DA further into the primary site, new interactions between DA and hDAT are formed. They find that EL2 and EL4 move closer into the vestibular binding site when both sites were occupied which then pushes the extracellular end of TM3 in on the vestibular binding site. This was observed to make I390 and F391 push the sidechain of W84 and L80 toward the primary binding site, in turn making Y156 and F320 rotate and push the DA molecule in the primary site further downward into its site. This rearrangement of residues causes L418 to interact with W84 instead of the ion in the Na2 site, and this switch is proposed to be relevant for ion release (Shan et al., 2011). For hSERT, MD simulations with substrate bound in both the primary and the vestibular site display unstable binding of 5-HT in the vestibular site (Koldsø et al., 2011). Furthermore, in the same study, a conformational change from outward- to inward-facing was observed only when a single substrate was bound in the central binding pocket, which does not favor a two-substrate allosteric mechanism. Clearly, the results of performing simulations with substrate in the vestibular binding site of hSERT and hDAT are not in agreement. Similarly, experiments on LeuT in regards to the two substrate mechanism has not yielded a clear answer either (Reyes and Tavoulari, 2011; Lim and Miller, 2012). The function of the vestibular site in MATs and other LeuT-fold transporters is thus still a subject of debate.

### Occlusion of the Central Binding Site from the Extracellular Side

**FIGURE 9:**
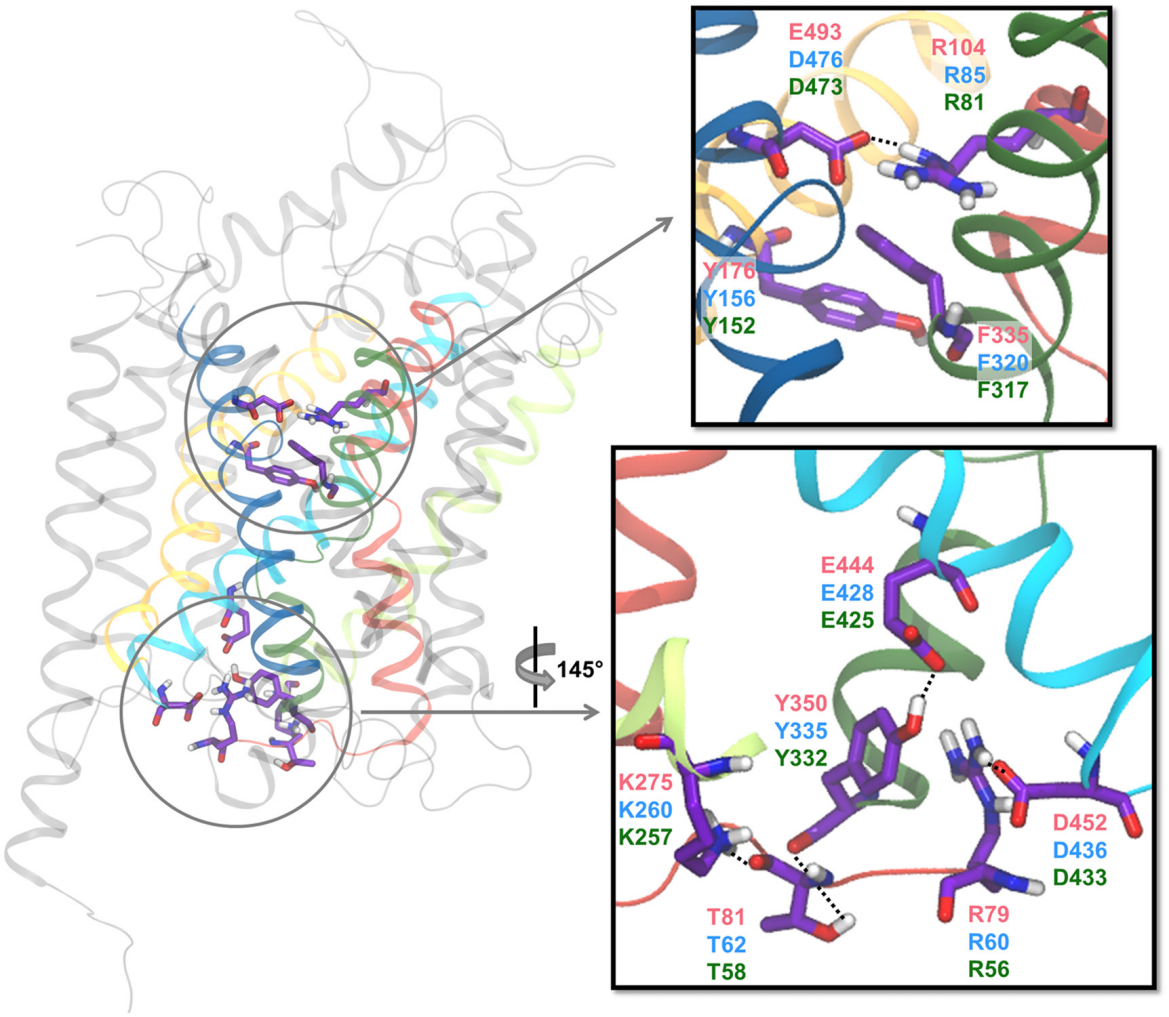
**Extracellular and intracellular gates.** The residues suggested to be involved in the extracellular and intracellular gating networks are shown in sticks with the carbon atoms in purple. In cartoon is shown TM1 (red), TM3 (yellow), TM5 (lime), TM6 (green), TM8 (cyan), and TM10 (blue). The residue indices are given in red for hSERT, blue for hDAT, and green for hNET. The figure is based on a homology model of hDAT (Koldsø et al., 2013a).

From electron paramagnetic resonance experiments it has been shown that LeuT without substrate and ions bound fluctuates between outward- and inward-facing conformations, and that the binding of Na^+^ stabilizes the outward open conformation, which is thought to trigger the binding of substrate (Claxton et al., 2010). In MD simulations it has been observed that when DA is bound in the primary binding site of rDAT or hDAT along with ions, the residues in the external gate (R85-D476 in hDAT) come together and form a salt bridge (Huang and Zhan, 2007; Huang et al., 2009; Gedeon et al., 2010; Stockner et al., 2013; **Figure 9**). On the other hand, MD simulations of rDAT without DA bound show no stable closure of the external gate which is congruent with the observation that the ions alone stabilize the outward open conformation of the transporter (Gedeon et al., 2010). Similar results have been observed for 5-HT binding to hSERT (Koldsø et al., 2011). A second extracellular gate has been described which consists of a hydrophobic lid directly above the primary binding site (Yamashita et al., 2005). In hDAT, the lid is formed by F320 and Y156, and in addition, the nearby residues F155 and Y84 have also been proposed to be part of this hydrophobic gate (Stockner et al., 2013). It has been observed that the average root-mean-square deviation (RMSD) of Cα atoms in loop segments change depending on whether or not DA is bound to rDAT during 15-20 ns MD simulations compared to the original conformation of the homology model used in the simulations (Gedeon et al., 2010). It was found that binding of DA increased the average RMSD of Cα atoms in a segment of EL2 by as much as 11 Å while lowering the RMSD of residue 512 in IL5 by almost 6 Å compared to simulations without DA. In fact, the segment of EL2 consisting of residues 186-203 (186-203 in hDAT) all had an average RMSD of at least 12 Å (Gedeon et al., 2010); however, this segment of EL2 was modeled *ab initio* due to the fact that the template, LeuT, has a shorter EL2 compared to the human MATs. The large RMSD values suggest that the modeled conformation of the segment is inaccurate, thus leading to artificially large movements during MD simulations. It was also observed that the N-terminal helical part of EL4, which is close to EL2, unwinds when DA is bound to rDAT during 30 ns MD simulations (Gedeon et al., 2010). A study by Stockner et al. (2013) found that it is possible to stabilize the movements of EL2 in hDAT by modeling hDAT while constraining the inducible Zn^2+^ site of hDAT. By creating a homology model using this restraint they observed that W184 anchors itself into the head groups of the extracellular leaflet, which in turn stabilizes the rest of EL2 in a conformation that is able to bind Zn^2+^ steadily for 200 ns, thus bringing the RMSD of EL2, with respect to the initial structure, down to a level comparable to the RMSD of the remaining loops. It is known that Zn^2+^ modulates hDAT by stabilizing the outward-facing conformation (Richfield, 1993) which suggests that the movement of EL2 is necessary for the conformational change from an extracellular to an intracellular facing conformation. In spite of this, solvation of the ion in the Na2 site from the intracellular side was observed along with closure of the extracellular gates (Stockner et al., 2013). Solvation of the Na2 site from the intracellular side has also been observed for hDAT in a study by Shan et al. (2011) using SMD (Shan et al., 2011), where they observe that a rotation of S262 and M424 is needed to enable the intracellular solvent to diffuse in to the Na2 site.

### Intracellular Opening and Release of Substrate and Ions

To allow the release of substrate and ions to the intracellular side, an intricate internal gating network has to open. Several gating systems have been proposed and different networks have been observed in crystal structures of LeuT and dDAT, however, it is difficult to determine which of them are actual gates and which are crystal artifacts, as well as their relevance for the human MATs. Several studies have found F76, F69, and F332 (Shan et al., 2011; Dehnes et al., 2014), and possibly also Y335 (Stockner et al., 2013; Dehnes et al., 2014) and W63 (Dehnes et al., 2014) in hDAT to form a hydrophobic cluster just below the primary binding site. By steering the substrate toward the cytoplasm in SMD simulations, it has been observed that these hydrophobic residues can rotate away from each other allowing for DA to move out of the binding site toward the cytoplasm (Shan et al., 2011; Dehnes et al., 2014). The rotation of F332 pushes F69 in TM1a resulting in TM1a moving away from TM6b and the center of the protein allowing for the next gating system to open. An intracellular gating network formed by several hydrogen bonds between residues has also been reported (Kniazeff et al., 2008) (see **Figure 9**). Guptaroy et al. (2009) report hydrogen bonding in hDAT between the backbone carbonyl of T62 and K260 and the sidechain hydroxyl group of T62 and the backbone carbonyl of Y335, which effectively links TM1 and TM6 together through interactions with the N-terminus, to be stable throughout 16 ns of MD simulation of hDAT in an outward-facing conformation. On the other hand, Dehnes et al. (2014) report stable hydrogen bonding between K65 and N340, thus linking TM1 to TM7, in MD simulations of the outward open hDAT during 5 ns. They also observe that this hydrogen bond is not present throughout 5 ns of simulation of hDAT in an inward open conformation (Dehnes et al., 2014). It should be noted that the just mentioned simulations are rather short (5–16 ns), which makes it difficult to asses the stability of the observed interactions on longer time scales. Another hydrogen bond between Y335 and E428 (in TM6 and TM8, respectively) has been reported to break due to rotation of Y335 when performing simulations of the conformational change from outward- to inward-facing hDAT using SMD (Shan et al., 2011). Unbiased MD simulations of hSERT similarly reveal that the interaction between the corresponding residues, namely Y350 and E444, is lost during the transition from outward-facing to inward-facing (Koldsø et al., 2011, 2013b). Additionally, the solvated N-terminal close to TM1a and the intracellular loop directly after TM8 (IL4) are also suggested to be connected by a salt bridge interaction. This salt bridge is present in most of the outward-facing crystal structures of dDAT (Penmatsa et al., 2013, 2015; Wang et al., 2015) and has also been observed to break during the transition toward the inward-facing conformation in MD simulations of both hDAT (R60 and D436) (Stockner et al., 2013) and hSERT (R79 and D452) (Koldsø et al., 2011). To allow complete release of substrate, this salt bridge most likely breaks, although it may be difficult to observe in MD simulations due to the common truncation of the terminals. In most MD studies the N-terminal is truncated shortly before the N-terminal arginine thus perhaps over-stabilizing the salt bridge by lack of alternative interactions by the N-terminal residues not included in the model.

Full release of substrate from either of the MATs has not yet been observed in equilibrium MD simulations. However, the conformational change from outward-facing to inward-facing has been observed in unbiased MD simulations of hSERT, followed by the release of Na^+^ from the Na2 site (Koldsø et al., 2011, 2013b). In all three MATs, the Na2 site contains an aspartate residue and for hSERT rotation of the side chain of this residue, D437, was observed to pull the ion out of the Na2 site and into the intracellular release pathway located between TM1, 5, 6, and 8 (Koldsø et al., 2011). Based on this observation, uptake experiments were performed with the D437N mutant. As expected, a large increase in the K_m_ value for Na^+^ was obtained for the mutant, stressing the importance of this residue for Na^+^ binding. On the other hand, similar values of K_m_ and V_max_ for 5-HT transport were obtained for wild type and D437N. Overall, this suggest that the Na^+^ ion in the Na2 site drives the transport of substrate, and that release of Na^+^ occurs prior to substrate release. Based on observations from MD simulations of hSERT, the conformational change between outward- and inward-facing can be described as a movement of the helices of the bundle with respect to the scaffold (Koldsø et al., 2011, 2013b), in accordance with the rocking bundle mechanism (Forrest et al., 2008). However, the simulations suggest that while the extracellular ends of helices in the bundle move in a concerted fashion, intracellular opening is mainly due to a hinge-type motion of TM1 and TM6, which have unwound central parts.

Release of DA to the cytoplasm has been observed to occur through two different paths in RAMD simulations of rDAT (Merchant and Madura, 2012); a pathway along TM6b and TM8 was observed to occur twice as often as the alternative pathway along TM1a and TM6b during 50 MD simulations of 0.1–0.7 ns duration. The latter pathway is similar to the one reported by Koldsø et al. (2011) for Na^+^ release. They also performed RAMD simulations of rDAT with DA occupying both the primary and the vestibular binding site, but were not able to determine conclusively if inward release of DA is improved by the vestibular site being occupied by DA since DA release was observed through four different pathways, and only a few times through each pathway. Shan et al. (2011) applied SMD to sample the inward release of DA, and found the exit pathway to be through the center of TM1, 5, 6, and 8, as both Koldsø et al. (2011) and Merchant and Madura (2012) have proposed, when alternating between SMD (2 ns intervals) and MD (4 ns intervals) simulations of hDAT (Dehnes et al., 2014). It was observed that rotamer changes in F76 and F322 allow DA to move from the primary binding site toward the intracellular milieu. The rotation of F332 provokes movement of F69 resulting in a significant movement of TM1a away from TM6b followed by breaking of a hydrogen bond between Y335 and E428 allowing continued movement of TM1a and the N-terminus away from TM6. They observe that water is able to move all the way to the primary binding site when simulations are performed with a DA molecule in each of the two sites whereas in simulations of hDAT with a single DA occupying the primary site, water is only able to access F332, which is approximately half way toward the primary binding site, (Shan et al., 2011; Dehnes et al., 2014). Thus the results indicate that substrate binding in the vestibular site may accelerate inward opening of hDAT. They argue that the DA molecule occupying the primary binding site increases its interaction with water when the vestibular site is also occupied due to a rotation of S262 and M424 that allows water to move toward the Na2 site from the intracellular side. Before DA is released to the cytoplasm they observe an interaction with E428 where DA is otherwise fully solvated and they propose this interaction to be the last stabilizing interaction between DA and hDAT before inward release.

## Conclusion

In this review we have demonstrated the progress in our understanding of MATs that has been based either directly or indirectly on MD simulations. Despite limitations due to force field accuracy and restricted sampling, the discussed studies show that MD simulations can shed light on the dynamic binding of substrates and ions as well as different types of inhibitors. Furthermore, it is possible to directly observe the initial steps of the mechanism of action of substrates and inhibitors in unbiased MD simulations, but also full release of substrate using enhanced sampling methods such as SMD or RAMD can be explored. It has been possible to observe both specific residue–residue interaction changes as well as large helix movements, e.g., when changing from an outward-facing conformation, through occluded states, until reaching an inward-facing conformation. In numerous cases, the results of MD studies have guided the design of new experiments and it is clear that combining MD simulations and biochemical experiments is beneficial for improving our understanding of both transporter action and inhibition. The established binding modes and behavior of neurotransmitters within MATs can greatly aid in structure-based development of new pharmaceutical drugs as well as in the ongoing optimization of existing drugs.

Only 8 years has passed since the first article containing an MD simulation of a MAT based on a LeuT-fold template was published (Yamashita et al., 2005), and the progress in sampling and force field accuracy has advanced enourmously since then (Weng and Wang, 2014; Bernardi et al., 2015; Perilla et al., 2015). Today, it is possible to calculate MD trajectories of biological systems of several μs per day on custom built supercomputers such as Anton (Dror et al., 2012), and advances in computer design has made μs time scales feasible even on ordinary computer clusters. Together with improved accuracy of force fields regarding proteins (Lindorff-Larsen et al., 2010; Best et al., 2012) as well as lipids (Klauda et al., 2010; Dickson et al., 2014) and ligands (Vanommeslaeghe et al., 2010; Mayne et al., 2013), the great progress that has been observed thus far is still only at the beginning stages of what will come in the future.

## Conflict of Interest Statement

The authors declare that the research was conducted in the absence of any commercial or financial relationships that could be construed as a potential conflict of interest.
